# Identification and Action Patterns of Two Chondroitin Sulfate Sulfatases From a Marine Bacterium *Photobacterium* sp. QA16

**DOI:** 10.3389/fmicb.2021.775124

**Published:** 2022-01-24

**Authors:** Lin Wei, Qingdong Zhang, Danrong Lu, Min Du, Xiangyu Xu, Wenshuang Wang, Yu-Zhong Zhang, Xunyi Yuan, Fuchuan Li

**Affiliations:** ^1^National Glycoengineering Research Center, Shandong Key Laboratory of Carbohydrate Chemistry and Glycobiology, NMPA Key Laboratory for Quality Research and Evaluation of Carbohydrate-Based Medicine, Shandong University, Qingdao, China; ^2^School of Life Sciences and Technology, Weifang Medical University, Weifang, China; ^3^College of Marine Life Sciences, Ocean University of China, Qingdao, China; ^4^Department of General Surgery, Qilu Hospital (Qingdao), Cheeloo College of Medicine, Shandong University, Qingdao, China

**Keywords:** marine bacterium, glycosaminoglycan, chondroitin sulfate/dermatan sulfate, sulfatase, substrate-degrading pattern

## Abstract

Chondroitin sulfate (CS)/dermatan sulfate (DS) is a kind of sulfated polyanionic, linear polysaccharide belonging to glycosaminoglycan. CS/DS sulfatases, which specifically hydrolyze sulfate groups from CS/DS oligo-/polysaccharides, are potential tools for structural and functional studies of CD/DS. However, only a few sulfatases have been reported and characterized in detail to date. In this study, two CS/DS sulfatases, PB_3262 and PB_3285, were identified from the marine bacterium *Photobacterium* sp. QA16 and their action patterns were studied in detail. PB_3262 was characterized as a novel 4-*O*-endosulfatase that can effectively and specifically hydrolyze the 4-*O*-sulfate group of disaccharide GlcUAβ1–3GalNAc(4-*O*-sulfate) but not GlcUAβ1–3GalNAc(4,6-*O*-sulfate) and IdoUAα1–3GalNAc(4-*O*-sulfate) in CS/DS oligo-/polysaccharides, which is very different from the identified 4-*O*-endosulfatases in the substrate profile. In contrast, PB_3285 specifically hydrolyzes the 6-*O*-sulfate groups of GalNAc(6-*O*-sulfate) residues located at the reducing ends of the CS chains and is the first recombinantly expressed 6-*O*-exosulfatase to effectively act on CS oligosaccharides.

## Introduction

Chondroitin sulfate (CS)/dermatan sulfate (DS), a class of natural polyanionic, linear and heterogeneous glycosaminoglycan (GAG), is ubiquitously distributed on the cell surface and the extracellular matrix (ECM) in animal tissues (Cohen and Merzendorfer, 2019). CS/DS chains link with core proteins to form proteoglycans, interact with a series of proteins, such as chemokines (Dyer et al., 2016; Sepuru and Rajarathnam, 2019), growth factors (Sirko et al., 2010; Zhang et al., 2019) or other ECM components, and participate in various physiological and pathological processes (Monneau et al., 2016; Jiang et al., 2019; Tighe and Garantziotis, 2019). The backbone of CS is composed of D-glucoronic acid (GlcUA) and *N*-acetyl-D-galactosamine (GalNAc) as the form of repeating disaccharide units GlcUAβ1–3GalNAc linked by β1–4 glycosidic bonds. Some D-GlcUA residues can also be epimerized into L-iduronic acid (IdoUA) residues under the catalysis of glucuronyl C5 epimerase to form a CS-DS hybrid chain (Mikami and Kitagawa, 2013). The backbone of CS/DS is usually decorated with differential sulfate groups at C2 of the GlcUA/IdoUA residue and C4 and C6 of the GalNAc residue by various specific sulfotransferases to produce various sulfated disaccharide units, such as the O unit (GlcUAβ1–3GalNAc), C unit [GlcUAβ1–3GalNAc(6S)], A unit [GlcUAβ1–3GalNAc(4S)], D unit [GlcUA(2S)β1–3GalNAc(6S)], and E unit [GlcUAβ1–3GalNAc(4S,6S)], where 2S, 4S, and 6S stand for 2-*O*-, 4-*O*-, and 6-*O*-sulfates, in CS as well as their IdoUA-containing counterparts in DS. The source of CS was found in both vertebrates and invertebrates, but their sulfation patterns are varying with the animal species (Vessella et al., 2019). Some terrestrial animal-derived CS preparations are almost only sulfated at C4/C6 of GalNAc residues, for example CS extracted from bovine and porcine trachea (Volpi et al., 2021). In contract, marine animals process a great deal of GAGs with some unusual sulfation patterns (Valcarcel et al., 2017). Some of them were oversulfated, such as E unit-rich CS-E from squid cartilage (Peng et al., 2021), iB unit [IdoUA(2S)α1-3GalNAc(4S)]-contained CS-B from sea squirt (Pavao et al., 1998), K unit [GlcUA(3S)β1-3GalNAc(4S)]-contained CS-K from octopus (Higashi et al., 2015), and S unit [GlcUA(2S,3S)β1-3GalNAc(4S)] or GlcUA(2S,3S)β1-3GalNAc(6S)-contained CS from the cephalothorax of pacific white shrimp (Cavalcante et al., 2018). These modifications make CS/DS chains highly microheterogeneous and complex biological macromolecules (Kusche-Gullberg and Kjellen, 2003).

CS/DS sulfatases, which specifically hydrolyze sulfate ester, release sulfate groups from CS/DS monosaccharide, disaccharide or/and oligo-/polysaccharide. CS/DS sulfatase belongs to the arylsulfatase family, their sequences, structures, and catalytic mechanisms are highly conserved in both eukaryotic and prokaryotic species, and all of the mature sulfatases contain a special α-formylglycine (FGly) residue that is decorated post-translationally (Hanson et al., 2004). Based on their substrate specificity, CS/DS sulfatases can be divided into Δ^4,5^hexuronate-2-*O*-sulfatase, *N*-acetylgalactosamine-4-*O*-sulfatase (GalNAc-4-*O*-sulfatase) and *N*-acetylgalactosamine-6-*O*-sulfatase (GalNAc-6-*O*-sulfatase); based on their degradation mode, they can also be also divided into exo-type CS/DS sulfatases, which act on the sulfate groups located at the ends of CS/DS chains, and endo-type CS/DS sulfatases, which can act on sulfate groups inside and outside the CS/DS chains (Wang et al., 2016). All of these sulfatases have the potential to become useful tools for structural and functional studies of CS/DS and the preparation of CS/DS oligo-/polysaccharides with specific sulfation patterns.

Accumulating evidence has proven that CS/DS sulfatases play significant roles in the metabolism and utilization of GAGs for microorganisms (Ndeh et al., 2020). However, only a few of them have been identified, including two endo-type GalNAc-4-*O*-sulfatases from *Bacteroides thetaiotaomicron* and *Vibrio* sp. FC509 (Ulmer et al., 2014; Wang et al., 2015a), two exo-type GalNAc-4-*O*-sulfatases from *Proteus vulgaris* and *Photobacterium* sp. FC615 (Yamagata et al., 1968; Wang et al., 2019b), two exo-type GalNAc-6-*O*-sulfatases from *Proteus vulgaris* and *Bacteroides thetaiotaomicron* (Yamagata et al., 1968; Ulmer et al., 2014), and three Δ^4,5^hexuronate-2-*O*-sulfatases from *Flavobacterium heparinum*, *Bacteroides thetaiotaomicron*, and *Photobacterium* sp. FC615 (Myette et al., 2003; Ulmer et al., 2014; Wang et al., 2019a), which cannot meet the requirements for research and application of highly complex CS/DS. For instance, the endo-type GalNAc-6-*O*-sulfatase remains to be identified thus far, and the only *N*-acetylgalactosamine-6-*O*-sulfatase that has been successfully recombinantly expressed can only act on 6-*O*-sulfataed GalNAc monosaccharide (Ulmer et al., 2014), although a 6-*O*-sulfatase isolated from *Proteus vulgaris* has been shown to act on 6-*O*-sulfated CS disaccharides (Yamagata et al., 1968). Recently, studies by our group and others have shown that marine bacteria are potentially rich sources of novel GAG-degrading enzymes with unique enzymatic properties (Han et al., 2014; Kurata et al., 2015; Wang et al., 2017; Peng et al., 2018; Zhang et al., 2020).

In this study, two novel CS sulfatases were identified from the marine bacterium *Photobacterium* sp. QA16 isolated from costal sediments (Zhang et al., 2020). One of them is an endo-type GalNAc-4-*O*-sulfatase, named PB_3262, and can hydrolyze the 4-*O*-sulfate groups inside the CS polysaccharides. Compared with the two identified 4-*O*-endosulfatases from *Bacteroides thetaiotaomicron* and *Vibrio* sp. FC509 (Ulmer et al., 2014; Wang et al., 2015a), PB_3262 shows a relatively high activity and specificity toward the monosulfated A unit, GlcUA-GalNAc(4S). The other is an exo-type GalNAc-6-*O*-sulfatase, named PB_3285, and can only hydrolyze the 6-*O*-sulfate groups located at the reducing ends of CS oligosaccharides, which is the first successfully recombinantly expressed exo-type 6-*O*-sulfatase acting on CS oligosaccharides rather than monosaccharides. The enzymatic characteristics and substrate degradation properties of these two enzymes were studied in detail. Moreover, the catalytic mechanisms of these two enzymes were preliminarily investigated via homology modeling and site-directed mutagenesis. The identification of these two enzymes will provide potential tools for the studies and applications of CS/DS.

## Materials and Methods

### Materials

The putative genes of two sulfatases, PB_3262 and PB_3285, were cloned from the genome of the marine bacterium *Photobacterium* sp. QA16 isolated from coastal sediments as described in a previous research (Table 1; Zhang et al., 2020). PrimeSTAR™ HS DNA polymerase for PCR amplification, restriction endonucleases and T4 ligase were purchased from Takara Inc. (Dalian, China). *Escherichia coli* competent BL21 cells (DE3) were purchased from Vazyme Biotech Co., Ltd. (Nanjing, China). The Genome Extraction Kit was purchased from Tiangen Biotech Co., Ltd. (Beijing, China). CS-A from bovine trachea, CS-C from shark cartilage, 2-aminobenzamide (2-AB), sodium cyanoborohydride (NaBH_3_CN) and commercial chondroitinase ABC (CSase ABC) from *Proteus vulgaris* were purchased from Sigma-Aldrich Inc., CS-D was extracted from shark cartilage (Nandini et al., 2005). CS-E was extracted from *Dosidicus gigas* cartilage (Peng et al., 2021). Porcine intestinal mucosa-derived DS was provided by Tiandong Pharma (Dongying, China). Unsaturated CS disaccharide standards were purchased from Iduron (Manchester, United Kingdom). Unsaturated disaccharides for reactions were prepared by separating the final products of CS-A, CS-C, CS-D, and CS-E digested by CSase ABC. Unsaturated CS tetrasaccharide ΔHexUA1–3GalNAc(6S)β1–4GlcUA(2S)β1–3GalNAc(6S) (ΔC-D) was prepared from the partial digest of CS-D by CSase ABC (Wang et al., 2017). Unsaturated CS tetrasaccharides ΔHexUA1–3GalNAc(6S)β1–4GlcUAβ1–3GalNAc(4S) (ΔC-A) and ΔHexUA(2S)1–3GalNAc(6S)β1–4GlcUAβ1–3GalNAc(6S) (ΔD-C) were isolated from the final products of CS-D digested with enCSase (Zhang et al., 2020). All of the tetrasaccharides were isolated and purified by anion-exchange chromatography and desalination with gel filtration chromatography HPLC, as described previously (Zhang et al., 2020).

**TABLE 1:** Strains, plasmids and primers of PB_3262 and PB_3285 used in this study.

Strains and plasmids	Description	Source
**Strains**		
*Photobacterium* sp. QA16	A GAG-degrading marine bacterium published in previous study	Zhang et al., 2020
*E. coli* BL21 (DE3)	F^–^ *omp*T *hsd*S (rB^–^, mB^–^) *gal dcm* (DE3)	Vazyme
**Plasmids**		
pET-30a	Expression vector; Kan^r^	Novagen
pET-30a-*pb_3262*	pET-30a carrying an *Nde*I-PB_3262-*Xho*I fragment encoding the recombinant protein of PB_3262 fused with a His_6_ tag at the C-terminus	This study
pET-30a-*pb_3285*	pET-30a carrying an *Nde*I-PB_3285-*Xho*I fragment encoding the recombinant protein of PB_3285 fused with a His_6_ tag at the C-terminus	This study
**Primers**		
PB_3262-F	5′-CATATGATGCCAGCTTGTAGCGCTG CCTCATC-3′	Sangon Biotech
PB_3262-R	5′-CTCGAGAGTGTAGGCATTCAGAA CACTGG-3′	
PB_3285-F	5′-CATATGCAAGGTACGGCAGAGCAA CCCAATG-3′	
PB_3285-R	5′-CTCGAGCTGTGCTGAGACCGTC AAGATC-3′	
PB_3262 C70A-F	5′-AACTTTCCACTCGCGACCCCCTTC CGCGGGATG-3′	
PB_3262 C70A-R	5′-GTCGCGAGTGGAAAGTTAGACACTG CCTGGGG-3′	
PB_3262 P72A-F	5′-ACTCTGTACCGCGTTCCGCGGGATG TTGATGA-3′	
PB_3262 P72A-R	5′-GGAACGCGGTACAGAGTGGAAAGTT AGACACTGC-3′	
PB_3262 R74A-F	5′-TTCGCGGGGATGTTGATGACCGGCC AGTACCC-3′	
PB_3262 R74A-R	5′-ATCAACATCCCCGCGAAGGGGGTA CAGAGTGGAAAGT-3′	
PB_3285 C109A-F	5′-GCCAGTGGCAGGCCCATCGCGCGCA GGCATGC-3′	
PB_3285 C109A-R	5′-ATGGGCCTGCCACTGGCGTGGCGA CATAGGCA-3′	
PB_3285 P111A-F	5′-AGTGTGTGGCGCGTCGCGCGCAG GCATGCTGA-3′	
PB_3285 P111A-R	5′-GCGACGCGCCACACACTGGCGTGG CGACATAG-3′	
PB_3285 R113A-F	5′-CCATCGGCGGCAGGCATGCTGACC GGACGTTT-3′	
PB_3285 R113A-R	5′-ATGCCTGCCGCCGATGGGCCACAC ACTGGCGT-3′	

### Sequence Analyses of Putative Sulfatase Genes *pb_3262* and *pb_3285*

The draft genome of *Photobacterium* sp. QA16 and genome annotation were performed as described in a previous study (Zhang et al., 2020). Sequence similarity analysis of these sulfatase sequences was performed using the Protein BLAST online^1^. The prediction of signal peptide was performed using the SignalP 4.0 server. The molecular mass of protein was estimated using Compute pI/MW tool on the ExPASy server of the Swiss Institute of Bioinformatics^2^. Multiple sequence alignment was performed using Bio-Edit (version 7.0.5.3) and the phylogenetic analysis was carried out using MEGA (version 7.0).

### Expression and Purification of Recombinant Sulfatases

The genes of putative sulfatases without signal peptides were individually amplified using PrimeSTAR™ HS DNA polymerase (Takara Inc., China) and their primers with *Nde*I/*Xho*I restriction enzyme sites (Table 1). The PCR products were inserted into the *Nde*I/*Xho*I restriction enzyme sites of pET-30a (+) with a His_6_ tag at the C-terminus of proteins. Recombinant expression plasmids (pET-30a-*pb_3262* and pET-30a-*pb_3285*) were transformed into *E. coli* BL21 (DE3) and fragment integrities were confirmed by DNA sequencing. To express the putative sulfatases, *E. coli* cells transfected with corresponding expression plasmid were cultured in 100 ml Luria-Bertani (LB) medium containing 50 μg/ml kanamycin at 37°C until the A_600_ values reached 0.8–1.0, and then induced with 0.05 mM isopropyl-1-thio-β-D-galactopyranosid (IPTG) at 16°C for 24 hours. The cells were collected by centrifugation and disrupted in ice-cold buffer A (50 mM Tris, 150 mM NaCl (pH 8.0)) by sonication (50 repetitions, 4 s). After centrifugation at 15,000 × g for 30 min at 4°C, the supernatants containing the soluble putative sulfatases were collected, filtrated and loaded on a nickel affinity column prepared with Nickel-Sepharose™ 6 Fast Flow (GE Healthcare, Sweden). The column was washed with buffer A containing 10 mM imidazole to remove the non-specifically bound impurities and then were eluted with buffer A with 250 mM imidazole to collect the target protein-contained fraction. After desalting with an Amicon Ultra 0.5-ml 10K unit (Millipore), the purity of protein in 50 mM Na_2_HPO_4_-NaH_2_PO_4_ (pH 7.5) containing 100 mM NaCl buffer was estimated by SDS-polyacrylamide gel electrophoresis (SDS-PAGE) followed by staining with Coomassie Brilliant Blue R-250. The concentrations of purified proteins were determined using a BCA Protein Assay Kit (Kangway, Shanghai).

### Specificity Assay of Recombinant Proteins Toward Various Chondroitin Sulfate Disaccharides and Polysaccharides

The substrate specificities of PB_3262 and PB_3285 were investigated by using four unsaturated CS disaccharides (2 nmol) with different sulfation patterns including ΔHexUA1–3GalNAc(4S) (ΔA), ΔHexUA1–3GalNAc(6S) (ΔC), ΔHexUA(2S)1–3GalNAc(6S) (ΔD) and ΔHexUA1–3GalNAc(4S,6S) (ΔE). Briefly, each disaccharide was treated with 2 μg of purified PB_3262 or PB_3285 and incubated with 50 mM Tris-HCl buffer (pH 7.0) at 25°C for 12 h. Disaccharides treated with inactivated corresponding enzymes were used as negative controls. The resulting mixtures were inactivated by boiling for 10 min and cooled on ice. After centrifugation, the supernatants were collected and 2-AB labeled as described in previous studies (Bigge et al., 1995; Kinoshita and Sugahara, 1999). The 2-AB-labeled samples were individually loaded on a YMC-Pack PA-G column (YMC, Japan) and eluted with a linear gradient of 16 to 460 mM NaH_2_PO_4_ over 60 min at 1.0 ml/min. The eluted fractions were monitored by fluorescent detector with excitation and emission wavelengths of 330 and 420 nm. The peaks of resulted disaccharides with specific sulfation patterns were assigned by comparing with 2-AB labeled authentic unsaturated CS disaccharide standards, and the relative content of the disaccharides was determined by calculating the area of the corresponding peaks.

Additionally, various CS/DS polysaccharides including CS-A, CS-E, DS, and CS-D were also treated with purified PB_3262 or PB_3285 to test their activity and specificity toward polysaccharides. Briefly, each polysaccharide (2 μg) was incubated with purified sulfatase (2 μg) at 25°C for 12 h. After boiling for 10 min and centrifugation at 15,000 × g, the supernatants were further treated with CSase ABC (5 mIU) at 30°C for 2 h. Then, the reaction system was inactivated by boiling once more and centrifuged to remove the denatured protein. The resultants were fluorescently labeled with 2-AB and analyzed by HPLC on YMC-Pack PA-G column as described above. The disaccharide compositions of the CS/DS polysaccharides used in this experiment were determined by digestion with CSase ABC followed by anion-exchange HPLC as described previously (Wang et al., 2015b).

### Biochemical Characterization of Recombinant Sulfatases PB_3262 and PB_3285

To determine the optimal temperatures of PB_3262 and PB_3285, their enzymatic activities were investigated by using 30 μg unsaturated disaccharides ΔA unit and ΔC unit as substrates, respectively, in 30 μl of 50 mM Tris-HCl buffer (pH 7.0) at temperatures from 0 to 70°C for 5 min. The effect of pH was investigated by using a series of buffers with various pH ranges pH 5.0–6.0, NaAc-HAc buffer (50 mM); pH 6.0–8.0, NaH_2_PO_4_-Na_2_HPO_4_ buffer (50 mM); and pH 7.0–10.0 Tris-HCl buffer (50 mM) in a total volume of 30 μl at the optimal temperature of corresponding enzyme for 5 min. At the same time, the activities of these two enzymes were also determined in imidazole (50 mM, pH 6.0–8.0), HEPES (50 mM, pH 7.0–8.0) and MES (50 mM, pH 5.0–6.0) buffers. The effects of chemicals (5 mM) on the enzymatic activities were evaluated at the optimal conditions (temperature and pH) of PB_3262 and PB_3285, respectively. Additionally, to determine the thermostability of these two sulfatases, purified enzymes were preincubated at temperatures from 0 to 40°C for 0–24 h, and the residual activities of PB_3262 and PB_3285 were measured by incubating the resulting enzymes with substrates ΔA and ΔC, respectively, for 5 min at their optimal conditions determined above. All reactions were carried out in duplicate, and the activities of enzymes were estimated by gel filtration HPLC on a Superdex™ Peptide 10/300 GL column eluted with 0.20 M NH_4_HCO_3_ at 0.4 ml/min. The eluted profile of the resultant was detected with a UV detector at 232 nm and analyzed using the LCsolution version 1.25 (Yamagata et al., 1968; Li et al., 2010).

The specific activity of PB_3262 to unsaturated CS disaccharide was measured by using 100 μg ΔA as substrate under its optimal conditions (50 mM Tris-HCl buffer, pH 7.0, 50°C). Aliquots (30 μl) were removed at various time intervals 0, 0.5, 1, and 2 min, and immediately denatured by boiling for 10 min. Similarly, the specific activities of PB_3285 against disaccharides ΔC, ΔD and ΔE were determined under optimal conditions (50 mM Tris-HCl buffer, pH 7.0, 30°C). To further determine the specific activity of PB_3262 against CS-A polysaccharide, CS-A (100 μg) was reacted with PB_3262 (500 μg) in 100 μl of 50 mM Tris-HCl buffer (pH 7.0) at 50°C, and a series of aliquots were taken and immediately denatured by boiling. After centrifugation, the supernatants were further digested with CSase ABC and the activities of enzymes were estimated by detecting the generation rate of desulfated disaccharides derived from disaccharide or polysaccharide substrates using gel filtration HPLC as described above. One unit of enzyme is defined as the amount of enzyme that hydrolyzes 1 μmol of sulfate groups in 1 min.

### Determination of the Action Pattern of 6-*O*-Exosulfatase PB_3285

The substrate-degrading mode of the strict exolytic sulfatase PB_3285 was estimated using two 6-*O*-sulfate-containing CS tetrasaccharides ΔC-D and ΔD-C as substrates. Each tetrasaccharide (0.5 nmol) was treated with PB_3285 (2 mU) at 25°C for 12 h to ensure the substrates were exhaustively degraded. The reacts were inactivated by boiling and digested with CSase ABC (5 mIU) at 30°C for 2 h. The final products were fluorescently labeled with 2-AB for anion exchange HPLC analysis as described above. Furthermore, a saturated trisaccharide GalNAc(6S)β1–4GlcUAβ1–3GalNAc was prepared from ΔC-A treated with glycosyl hydrolase, which was used to remove the ΔHexUA residue from the non-reducing end of CS chain (Mori et al., 2003), and PB_3262, which was used to hydrolyze 4-*O*-sulfate group of GalNAc(4S) residue located at the reducing end. The saturated trisaccharide (0.5 nmol) was treated with PB_3285 followed by labeling with 2-AB and HPLC analysis for investigating if PB_3285 hydrolyzes the 6-*O*-sulfate group of GalNAc(6S) residue located at the non-reducing end of CS oligosaccharide.

### Catalytic Mechanism Analysis of PB_3262 and PB_3285

To explore the probable catalytic mechanisms of PB_3262 and PB_3285, homology modeling of the two enzymes was carried out using SWISS-MODEL software^3^. Structurally identified chondroitin sulfate/dermatan sulfate 4-*O*-endosulfatase protein (endoVB4SF) (PDB code: 6J66) (Wang et al., 2019b) and *N*-acetylgalactosamine-6-sulfatase (GalN6S) (PDB code: 4FDI) (Rivera-Colon et al., 2012) were used as templates for PB_3262 and PB_3285, respectively. Furthermore, based on the modeling structures of PB_3262 and PB_3285 a series of potential active site residues were predicted and individually mutated to alanine (Ala) via site-directed mutagenesis using a Fast Mutagenesis Kit V2 (Vazyme Biotech Co., Ltd., Nanjing). Primers used for mutants were list in Table 1. The mutants were expressed and their activities were individually estimated using ΔA and ΔC as substrates followed by analyzing with gel filtration HPLC as described for the wild enzymes above.

## Results

### Gene and Protein Sequences of Two Sulfatases

The length of the putative CS/DS 4-*O*-endosulfatase PB_3262 gene (GenBank™ accession number: MZ358908) is 1,488 bp, in which the GC content is 50.47%. This gene encodes a protein containing 495 amino acid residues and the protein has a theoretical molecular weight of 56.7 kDa and a theoretical isoelectric point of 6.16. SignalP 4.0 server analysis shows that PB_3262 does not possess an *N*-terminal signal peptide. A BLASTp search showed that PB_3262 shared the highest sequence identity (85.51% identity under 97% query cover) with an identified exo-type 4-*O*-sulfatase exoPB4SF from *Photobacterium* sp. FC615 (Wang et al., 2019b) and a second highest sequence identity with another endo-type GalNAc-4-*O*-sulfatase endoVB4SF from *Vibrio* sp. FC509 (82.66% identity under the 99% query cover) (Wang et al., 2015a). Additionally, the putative CS 6-*O*-exosulfatase PB_3285 gene (GenBank™ accession number: MZ358909) is 1,608 bp in length with a GC content of 53.98%, which encodes a protein with 507 amino acid residues, a molecular mass 57.3 kDa, an isoelectric point of 5.55, and a signal peptide composed of 28 amino acid residues. According to the BLASTp search, PB_3285 shares relatively lower sequence identity (27.82% identity under the 69% query cover) with an identified GalNAc-6-*O*-sulfatase from *Bacteroides thetaiotaomicron* (Ulmer et al., 2014).

**FIGURE 1:**
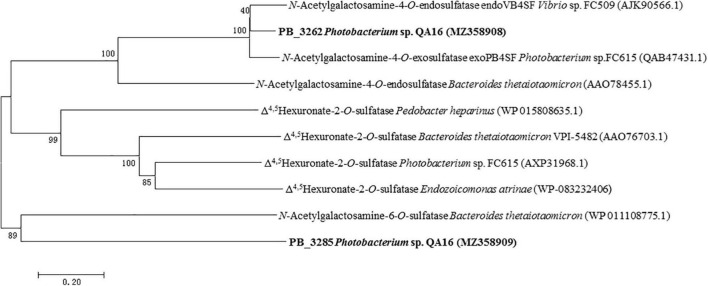
Phylogenetic analysis of PB_3262 and PB_3285. Phylogenetic analysis of PB_3262 and PB_3285 was executed based on Clustal W multiple alignments with identified CS/DS sulfatases from bacteria. The phylogenetic tree was built with MEGA version 7.0.26 software using the neighbor-joining algorithm. The percentage of replicate trees in which the associated taxa clustered together in the bootstrap test (1,000 replicates) is shown next to the branches.

The phylogenetic analysis also shows that PB_3262 clusters into the GalNAc 4-*O*-sulfatase clade and PB_3285 clusters with GalNAc 6-*O*-sulfatase, so we preliminarily speculate that PB_3262 is a CS/DS GalNAc-4-*O*-sulfatase and PB_3285 is a CS GalNAc-6-*O*-sulfatase (Figure 1).

### Heterologous Expression and Purification of PB_3262 and PB_3285 in *E. coli*

**FIGURE 2:**
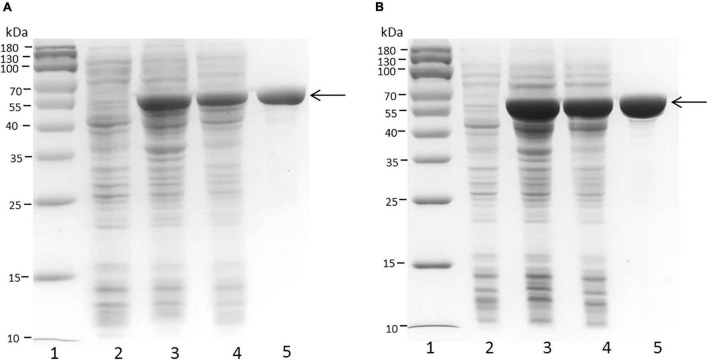
SDS-PAGE of crude and purified recombinant proteins PB_3262 and PB_3285 expressed in *E. coli*. Expression and purification of recombinant proteins PB_3262 **(A)** and PB_3285 **(B)** were analyzed by SDS-PAGE using 13.2% polyacrylamide gels. lane 1, Prestained Protein Marker (MP102) (Vazyme); lane 2, lysate of cells transfected with empty plasmid (pET-30a); lane 3, lysate of IPTG-induced cells transfected with expression plasmid; lane 4, lysate supernatant of induced cells with expression plasmid; lane 5, recombinant PB_3262 or PB_3285 purified with Ni^2+^ affinity chromatography. Molecular mass markers and their corresponding masses are also indicated.

The full-length sequences of the PB_3262 and PB_3285 genes without the signal peptide were amplified from the genomic DNA of *Photobacterium* sp. QA16, cloned into the expression vector pET-30a and expressed in *E. coli* BL21 (DE3) cells as described in section “Materials and Methods.” The SDS-PAGE results showed that both PB_3262 and PB_3285 could be successfully expressed mainly in soluble protein (approximately 0.5 g/L and 1 g/L, respectively) with the correct molecular mass (Figure 2). The recombinant proteins PB_3262 and PB_3285 were further purified by Ni-NTA affinity chromatography to approximately 95% purity. The expression and purification data of two enzymes are shown in Supplementary Table 1.

### Specificity of PB_3262 and PB_3285 Toward Chondroitin Sulfate Disaccharides

**FIGURE 3:**
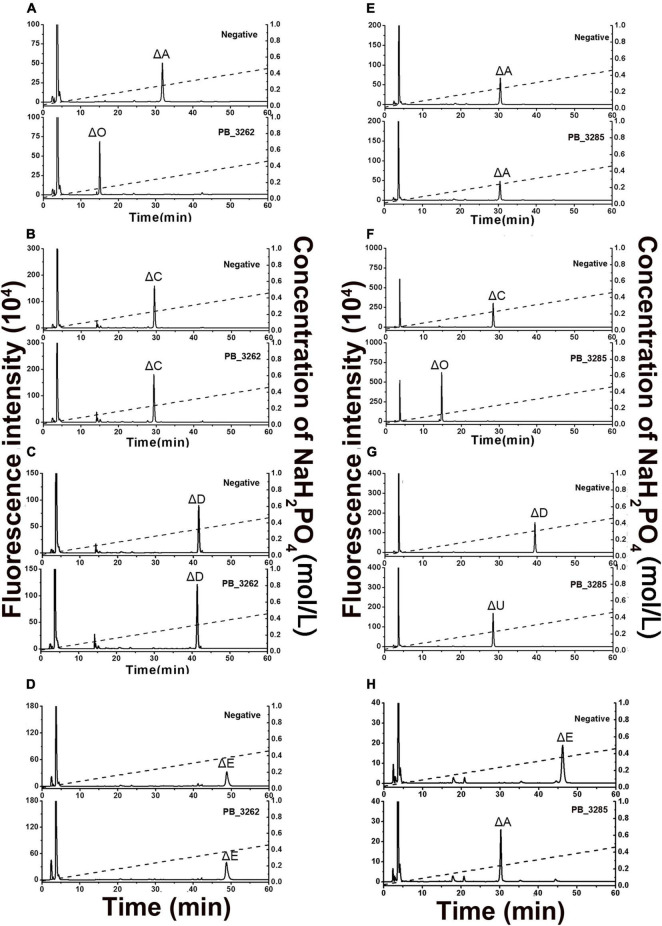
Product analysis of various unsaturated CS disaccharides treated with PB_3262 and PB_3285. Unsaturated CS disaccharides ΔA **(A,E)**, ΔC **(B,F)**, ΔD **(C,G)** and ΔE **(D,H)** were treated with inactivated (top) or original (bottom) PB_3262 **(A–D)** and PB_3285 **(E–H)**. All of the products were labeled with 2-AB and then analyzed using anion-exchange HPLC as described under section “Materials and Methods.” The elution positions of the standard disaccharides are indicated: ΔO (ΔHexUA1–3GalNAc), ΔA [ΔHexUA1–3GalNAc(4S)], ΔC [ΔHexUA1–3GalNAc(6S)], ΔD [ΔHexUA(2S)1–3GalNAc(6S)], ΔE [ΔHexUA1–3GalNAc(4S,6S)] and ΔU [ΔHexUA(2S)1–3GalNAc]. The peaks detected around 3 min in the figures are derived from fluorescent labeling reagents.

The activities and specificities of these two putative sulfatases were estimated by using various unsaturated CS disaccharide with specific sulfation patterns as substrates. The results showed that the putative GalNAc-4-*O*-sulfatase PB_3262 hydrolyzed the 4-*O*-sulfate group in ΔA to produce unsulfated disaccharide ΔHexUA1–3GalNAc (ΔO) but did not affect the 6-*O*-sulfate groups in ΔC and ΔD or the 2-*O*-sulfate group in ΔD (Figures 3A–C). Notably, the activity of PB_3262 toward 4-*O*-sulfate containing disulfated disaccharide ΔE was hardly detected (Figure 3D), which is significantly different from the identified GalNAc-4-*O*-sulfatases BT_3349, exoPB4SF and endoVB4SF with broader substrate selectivity (Ulmer et al., 2014; Wang et al., 2015a, 2019b). These results indicate that PB_3262 is a quite strict GalNAc-4-*O*-sulfatase to monosulfated A unit compared with those identified CS/DS 4-*O*-sulfatases. In contrast, PB_3285 does not affect the sulfation of ΔA but is capable of acting on the 6-*O*-sulfate group-containing disaccharide ΔC, ΔD, and ΔE to produce ΔO, ΔU [ΔHexUA(2S)1–3GalNAc] and ΔA (Figures 3E–H), and thus belongs to a typical GalNAc-6-*O*-sulfatase. In addition, these two enzymes could not act on heparan sulfate (HS) disaccharides with different sulfation patterns (Supplementary Figure 1), indicating a strict substrate specificity of these CS/DS sulfatases.

### Biochemical Characteristics of PB_3262 and PB_3285

**FIGURE 4:**
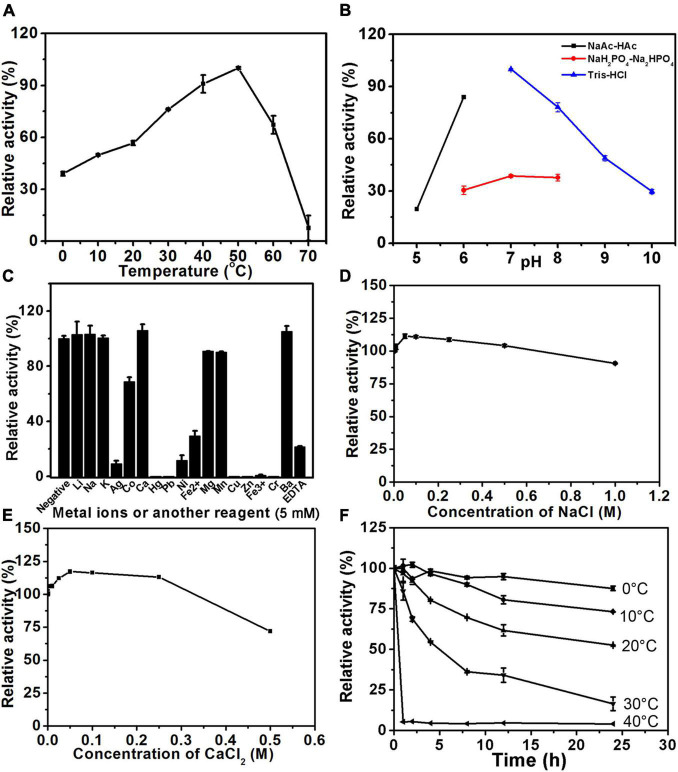
Biochemical characteristics of PB_3262. The biochemical characteristics of PB_3262 were investigated using disaccharide ΔA (1 mg/ml) as substrate as described under section “Materials and Methods.” **(A)** The effects of temperature on the activity of PB_3262 were determined at 0–70°C and the activities were presented as relative values calculated by comparing with the highest (100%) obtained at 50°C. **(B)** The effects of pH were determined in buffers (NaAc-HAc buffer, NaH_2_PO_4_-Na_2_HPO_4_ buffer and Tris-HCl buffer) with different pH values from 5 to 10 and the activities were presented as relative values calculated by comparing with the highest obtained in the 50 mM Tris-HCl buffer (pH 7.0). **(C)** The effects of metal cations were measured in Tris-HCl buffer (pH 7.0) containing 5 mM various metal ions and the activities were presented as relative values calculated by comparing with the highest obtained in Tris-HCl buffer (pH 7.0) only. **(D)** The thermostability of PB_3262 was estimated by preincubating this enzyme in 50 mM Tris-HCl buffer (pH 7.0) at temperatures from 0 to 70°C for 0–24 h followed by measuring residual activities. **(E)** The effects of Na^+^ concentration on the activity of PB_3262 were assayed in Tris-HCl buffer (pH 7.0) containing 0–1 M NaCl and the activities were presented as relative values calculated by comparing with that obtained in the buffer without Na^+^. **(F)** The effects of Ca^2+^ concentration were measured in Tris-HCl buffer (pH 7.0) containing 0–0.5 M CaCl_2_ and the activities were presented as relative values calculated by comparing with that obtained in the buffer without Ca^2+^. Error bars represent means of duplicates ± S.D.

The biochemical characteristics of PB_3262 and PB_3285, including their optimal temperature and pH, the effects of chemicals on their activities, and their thermostability, were investigated by using ΔA and ΔC as substrates, respectively. The optimal pH of the 4-*O*-sulfatase PB_3262 is 7.0 and it shows a maximum reaction rate in 50 mM Tris-HCl buffer (pH 7.0) (Figure 4B and Supplementary Figure 2A). In this buffer, PB_3262 exhibits the maximal rate at 50°C and a relatively high activity (> 60%) in the range of 30–60°C (Figure 4A). Additionally, divalent metal ions Ca^2+^ and Ba^2+^ exhibit slightly enhancing effect on the activity of PB_3262, which is consistent with the observation that the divalent metal ion chelating agent EDTA strongly inhibits the enzyme activity, and on the contrary some test metal ions, such as Ag^+^, Hg^2+^, Pb^2+^, Cu^2+^, Zn^2+^, Fe^3+^, and Cr^3+^, strongly inhibit the enzyme activity (Figure 4C). Furthermore, the optimal concentrations of both Na^+^ and Ca^2+^ were 50 mmol/L (Figures 4D,E).

**FIGURE 5:**
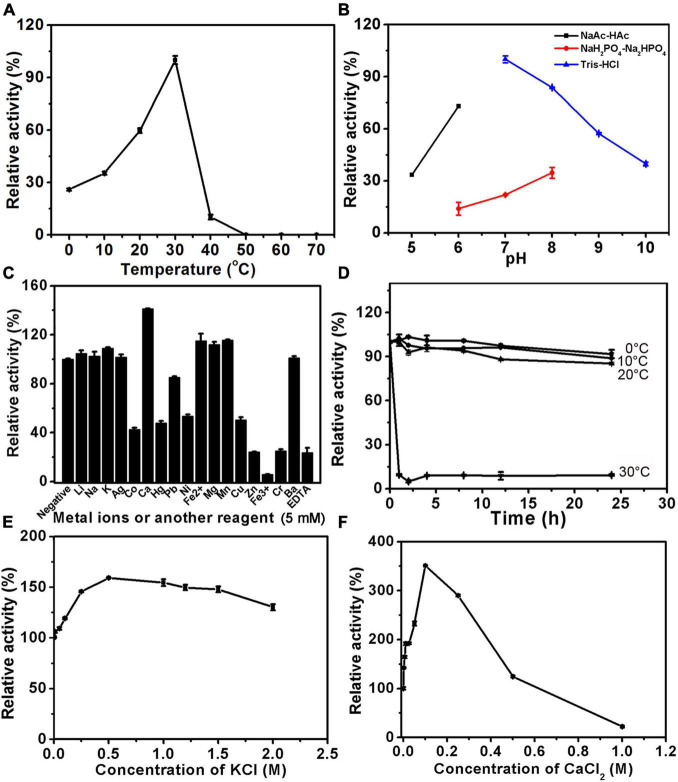
Biochemical characteristics of PB_3285. The biochemical characteristics of PB_3285 were investigated using disaccharide ΔC (1 mg/ml) as substrate as described under section “Materials and Methods.” **(A)** The effects of temperature on the activity of PB_3285 were determined at 0–70°C and the activities were presented as relative values calculated by comparing with the highest (100%) obtained at 30°C. **(B)** The effects of pH were determined in buffers (NaAc-HAc buffer, NaH_2_PO_4_-Na_2_HPO_4_ buffer and Tris-HCl buffer) with different pH values from 5 to 10 and the activities were presented as relative values calculated by comparing with the highest obtained in the 50 mM Tris-HCl buffer (pH 7.0). **(C)** The effects of metal cations were measured in Tris-HCl buffer (pH 7.0) containing 5 mM various metal ions and the activities were presented as relative values calculated by comparing with the highest obtained in Tris-HCl buffer (pH 7.0) only. **(D)** The thermostability of PB_3285 was estimated by preincubating this enzyme in 50 mM Tris-HCl buffer (pH 7.0) at temperatures from 0 to 70°C for 0–24 h followed by measuring residual activities. **(E)** The effects of K^+^ concentration on the activity of PB_3285 were assayed in Tris-HCl buffer (pH 7.0) containing 0–2 M KCl and the activities were presented as relative values calculated by comparing with that obtained in the buffer without K^+^. **(F)** The effects of Ca^2+^ concentration were measured in Tris-HCl buffer (pH 7.0) containing 0–1 M CaCl_2_ and the activities were presented as relative values calculated by comparing with that obtained in the buffer without Ca^2+^. Error bars represent means of duplicates ± S.D.

In the case of 6-*O*-sulfatase PB_3285, it has the maximal catalytic rate at 30°C but rapidly loses its activity when the temperature is lower or higher than 30°C (Figure 5A). Similar to PB_3262, PB_3285 has the optimum pH of 7.0 and a maximum reaction rate in 50 mM Tris-HCl buffer (Figure 5B and Supplementary Figure 2B). Interestingly, unlike PB_3262, the activity of PB_3285 was strongly promoted by 100 mM Ca^2+^ (350%) and 500 mM K^+^ (160%) and showed some resistance to heavy metal ions such as Co^+^, Hg^2+^, Ni^2+^ and Cu^2+^ (Figures 5C–E). EDTA also strongly inhibited the activity of this enzyme (Figure 5C), confirming the key role of Ca^2+^ in the catalytic mechanism. Notably, PB_3285 shows more halophilic properties than PB_3262, which may be due to the difference in their location in the cell.

Furthermore, the thermostabilities of PB_3262 and PB_3285 were investigated under their optimal conditions after incubation at a series of temperatures for 0–24 h. The results showed that both enzymes were unstable with the long incubation over 20°C (Figures 4F, 5F). At 30°C, the activity of PB_3262 slowly decreased to less than 20% within 24 h and that of PB_3285 was almost lost within 1 h, indicating that both enzymes are temperature sensitive and cannot tolerate high temperatures. Considering that both enzymes have relatively good stability and specific activity below 30°C (Figures 4A,F, 5A,F), instead of their optimal temperatures 25°C was chosen as the reaction temperature in the following experiments of the two enzymes.

The specificity activities of 4-*O*-endosulfatase PB_3262 were determined by using ΔA as the substrate under the optimal conditions. The specific activity of PB_3262 toward the ΔA is 165.75 U/mg, which is higher than those of PB4SF and VB4SF identified in previous studies (Wang et al., 2015a, 2019b). The specific activities of 6-*O*-exosulfatase PB_3285 against various unsaturated 6-*O*-sulfated CS disaccharides were also measured under its optimum conditions. The results show that the activities of PB_3285 toward ΔC, ΔD and ΔE are 3.64, 0.15 and 0.59 mU/mg, respectively, which are much lower than those of PB_3262 (Table 2).

**TABLE 2:** Specific activities of PB_3262 and PB_3285.

	Disaccharide				Polysaccharide
	ΔA unit (U/mg)	ΔC unit (mU/mg)	ΔD unit (mU/mg)	ΔE unit (mU/mg)	CS-A (mU/mg)
PB_3262	165.75 ± 11.75	N.D.	N.D.	N.D.	146.71 ± 3.32
PB_3285	N.D.	3.64 ± 0.68	0.15 ± 0.02	0.59 ± 0.05	N.D.

*Enzyme activities were expressed as average value ± standard deviation.*

*N.D. means not detected.*

### Activities of PB_3262 and PB_3285 Toward Chondroitin Sulfate/Dermatan Sulfate Polysaccharides

**FIGURE 6:**
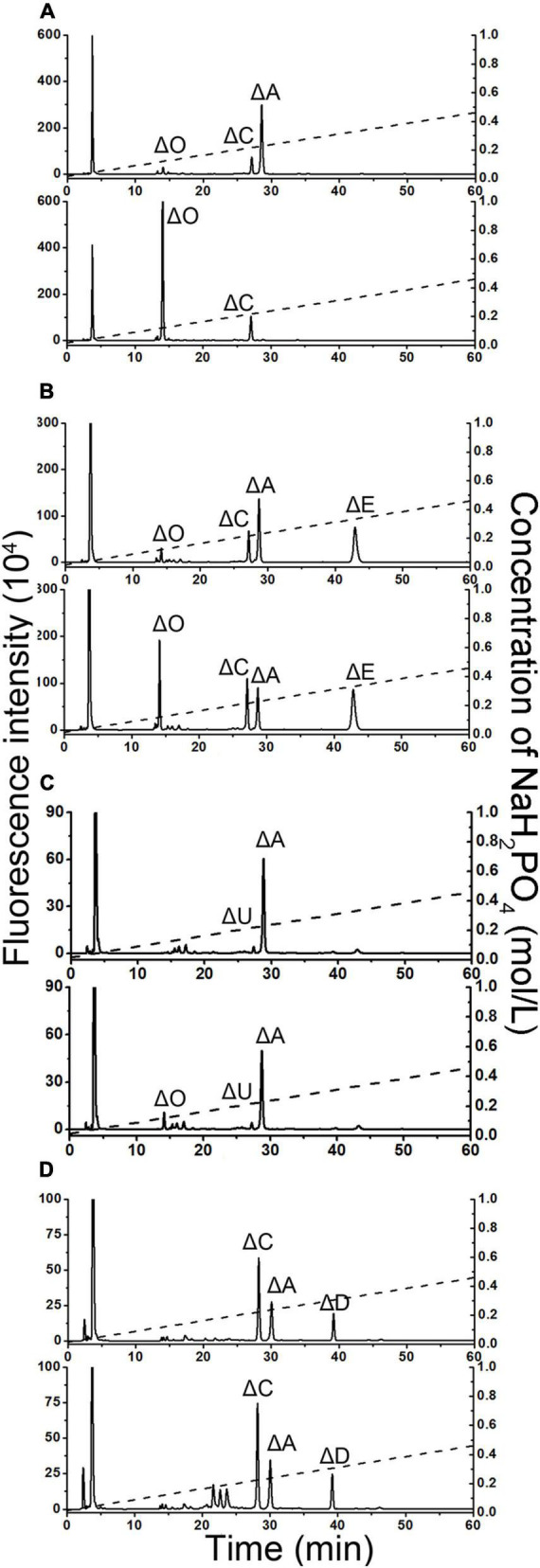
Disaccharide analysis of various 4-*O*-sulfated or 6-*O*-sulfated CS polysaccharides treated with PB_3262 and PB_3285. Three types of 4-*O*-sulfate-containing polysaccharides CS-A **(A)**, CS-E **(B),** and DS **(C)** were hydrolyzed without (top) or with (bottom) PB_3262 at 25°C for 12 h as described in section “Materials and Methods,” and the 6-*O*-sulfate-containing polysaccharide CS-C **(D)** was treated without (top) or with (bottom) PB_3285 under the same conditions. The resultants were digested with CSase ABC and labeled with 2-AB for disaccharide analysis by anion-exchange HPLC as mentioned above. The elution positions of the standard disaccharides are indicated: ΔO (ΔHexUA1–3GalNAc), ΔA [ΔHexUA1–3GalNAc(4S)], ΔC [ΔHexUA1–3GalNAc(6S)], ΔD [ΔHexUA(2S)1–3GalNAc(6S)], ΔE [ΔHexUA1–3GalNAc(4S,6S)] and ΔU [ΔHexUA(2S) 1–3GalNAc]. The peaks detected around 3 min in the figures are derived from fluorescent labeling reagents.

To test whether PB_3262 and PB_3285 are capable of acting on polysaccharides, CS/DS polysaccharides containing 4-*O*-sulfate groups such as CS-A, which is rich in A unit (76%), CS-E, which contains disulfated E unit (36%), and DS, in which D-GlcUA residues are epimerized to L-IdoUA and thus is rich in IdoUAα1–3GalNAc(4S) (iA unit) (88%), were used as the substrates of PB_3262, and CS-D, which is rich in 6-*O*-sulfate containing monosulfated C unit (49%) and disulfated D unit (18%), was used as the substrate of PB_3285 (Figure 6 and Supplementary Table 2). The disaccharide analysis showed that after treatment with PB_3262, the peak of ΔA detected in untreated CS-A disappeared completely, and correspondingly, the peak of ΔO increased sharply (Figure 6A), indicating that the A units were completely transformed to the O unit by 4-*O*-desulfation with this enzyme. In contrast, only trace amounts of E and iA units in CS-E and DS can be 4-*O*-desulfated to form C and iO units by this enzyme, respectively (Figures 6B,C). These results suggest that PB_3262 is an efficient endo-type GalNAc-4-*O*-sulfatase with high selectivity to hydrolyze 4-*O*-sulfate groups from mono-sulfated A units but not disulfated E and IdoUA-containing iA units in CS/DS. Additionally, PB_3285 cannot act on the 6-*O*-sulfate-containing C and D units in CS-D polysaccharide (Figure 6D), suggesting that it is an exo-type GalNAc-6-*O*-sulfatase.

The specificity activities of 4-*O*-endosulfatase PB_3262 toward CS-A polysaccharide were also determined by using CS-A polysaccharide as the substrates under the optimal conditions. The results showed that the specific activity (146.71 mU/mg) was a thousand times lower than that toward unsaturated A unit disaccharide (165.75 U/mg) (Table 2), indicating that PB_3262 prefers to act on low molecular substrates similar to the case of a 4-*O*-endosulfatase from *Vibrio* sp. FC509 (Wang et al., 2015a).

### Determination of the Action Pattern of 6-*O*-Exosulfatase PB_3285

**FIGURE 7:**
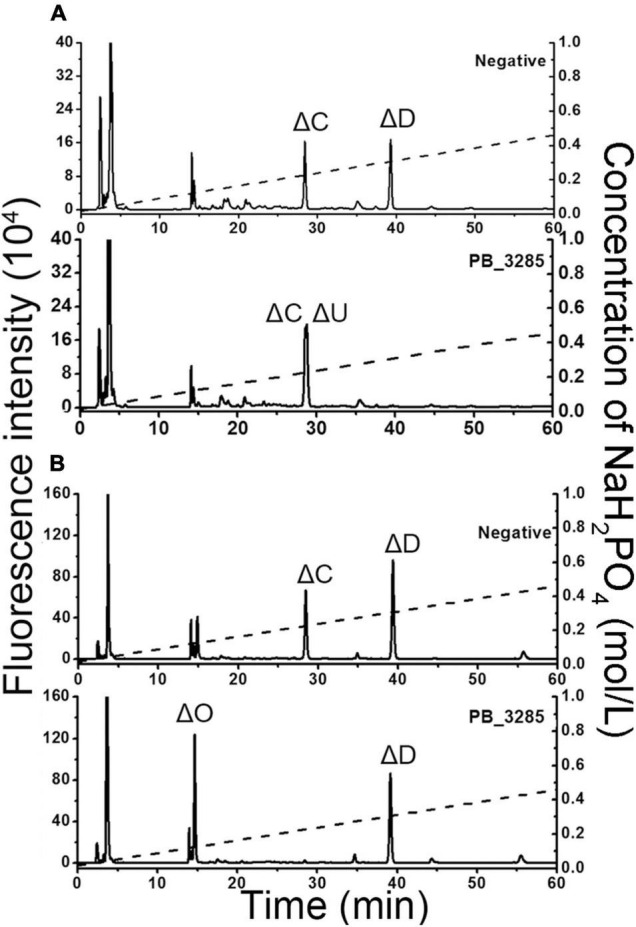
Determination of the catalytic site of 6-*O*-exosulfatase PB_ 3285 on the unsaturated tetrasaccharides substrates. Two tetrasaccharides ΔC-D **(A)** and ΔD-C **(B)** were treated without (top) or with (bottom) 6-*O*-exosulfatase PB_3285. After degradation with CSase ABC, the disaccharide composition of each tetrasaccharide was analyzed by anion-exchange HPLC as described in section “Materials and Methods.” The elution positions of the standard disaccharides are indicated: ΔO (ΔHexUA1–3GalNAc), ΔC [ΔHexUA1–3GalNAc(6S)], ΔD [ΔHexUA(2S)1–3GalNAc(6S)] and ΔU [ΔHexUA(2S)1–3GalNAc]. The peaks detected around 3 min in the figures are derived from fluorescent labeling reagents.

As described above, GalNAc-6-*O*-sulfatase PB_3285 possesses an exo-type catalytic manner. To further elucidate the substrate-degrading pattern of PB_3285, two structure-defined unsaturated 6-*O*-sulfated CS tetrasccharides, ΔC-D [ΔHexUA1–3GalNAc(6S)β1–4GlcUA(2S)β1–3GalNAc (6S)] and ΔD-C [ΔHexUA(2S)1–3GalNAc(6S)β1–4GlcUAβ1–3GalNAc(6S)], were individually hydrolyzed by PB_3285, and then the disaccharide compositions of the resultants were analyzed as described under section “Materials and Methods.” The results showed that the D unit located at the reducing end of ΔC-D was transferred in to the U unit but the C unit at the non-reducing end was not affected by this enzyme (Figure 7A). Additionally, the reducing end C unit of ΔD-C was correspondingly hydrolyzed to the O unit, but the non-reducing end D unit was unchanged (Figure 7B). Moreover, a saturated trisaccharide GalNAc(6S)β1–4GlcUAβ1–3GalNAc, which contains a non-reducing end GalNAc(6S), was prepared by digesting ΔC-A with glycosyl hydrolase and PB_3262, and treated with PB_3285. Results showed that PB_3285 is unable to hydrolyze the 6-*O*-sulfate group located at the non-reducing end of GalNAc(6S)β1–4GlcUAβ1–3GalNAc (Supplementary Figure 3E), indicating that the unsaturated ΔHexUA residue is not a key factor to affect the substrate-degrading direction of this enzyme. Taken together, these results suggest that PB_3285 is an exolytic sulfatase that strictly hydrolyzes the 6-*O*-sulfate esters of GalNAc residues located at the reducing end of CS chains.

### Tertiary Structure Modeling and Key Catalytic Sites of PB_3262 and PB_3285

**FIGURE 8:**
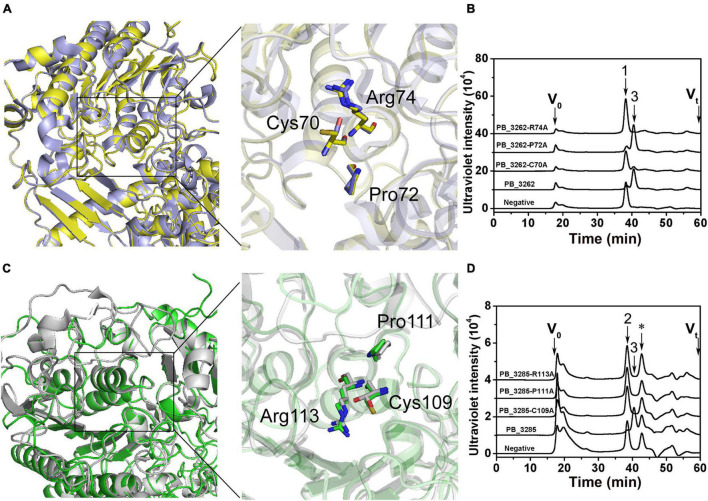
Tertiary structure modeling, structural alignment and site direct mutants of PB_3262 and PB_3285. Structural superposition of PB_3262 (*yellow*) to endoVB4SF (*cyans*) and PB_3285 (*green*) to GalN6S (*gray*). The homology modeling of PB_3262 **(A)** and PB_3285 **(C)** was predicted using SWISS-MODEL software online, and the conserved residues in active sites are shown with sticks structure. The conserved residues of PB_3262 **(B)** and PB_3285 **(D)** were individually replaced with Ala by site-directed mutagenesis. The activities of the mutants against ΔA **(B)** and ΔC **(D)** were estimated by gel filtration HPLC on a Superdex Peptide column at 232 nm. V_0_, void volume; V_t_, total volume. The elution positions of unsaturated CS disaccharides are indicated as follows: 1, ΔA; 2, ΔC; and 3, ΔO. *, salt from reaction buffer.

To preliminarily interpret the catalytic mechanisms of these two sulfatases, the three-dimensional structures of PB_3262 and PB_3285 were constructed with SWISS-MODEL homology modeling by using their homologous enzyme endo-type 4-*O*-sulfatase endoVB4SF from *Vibrio* sp. FC509 (82.66% identity under the 99% query cover) and lysosome *N*-acetylgalactosamine-6-sulfatase GalN6S (27.89% identity under the 89% query cover) as templates, respectively. Based on the molding structures, three amino acid residues are highly conserved in these two enzymes and their templates, including Cys70, Pro72 and Arg74 in PB_3262 (Figure 8A) and Cys109, Pro111, and Arg113 in PB_3285 (Figure 8C), indicating that they should play key roles in the catalytic mechanisms of these enzymes. To confirm this speculation, these conserved residues were individually muted to Ala by site-directed mutagenesis. The results showed that the activities of mutants PB_3262-C70A and PB_3262-R74A against ΔA were almost completely lost, but that of mutant PB_3262-P72A did not change significantly compared with the wild type (Figures 8B,D). In contrast, three mutants C109A, P111A, and R113A of PB_3285 were inactivated. These results suggest that these highly conserved residues should play important but different roles in the catalytic mechanisms of different CS/DS sulfatases.

## Discussion

Bacterial CS/DS sulfatases, an essential participator of CS/DS metabolism, play key roles in bacteria utilizing complex GAGs as a nutrient source (Ndeh et al., 2020). Owing to their properties of specifically hydrolyzing sulfate groups on CS/DS oilgo-/polysaccharides, CS/DS sulfatases have become potential tools for editing the sulfation patterns of CS/DS chains in related structure-function studies and oligosaccharide preparation. However, only a few bacterial sulfatases have been studied in detail. In this study, two CS sulfatases with unique enzymatic properties were identified from the genome of the marine bacteria *Photobacterium* sp. QA16. The endo-type 4-*O*-sulfatase PB_3262 exhibits efficient endolytic activity and high specificity toward monosulfated A units but not disulfated E units and IdoUA-containing iA units in CS/DS polysaccharides. Strictly exolytic GalNAc-6-*O*-sulfatase PB_3285 specifically removes all of the 6-*O*-sulfate groups on the reducing end GalNAc residues of CS chains. Both enzymes are temperature sensitive and halophilic to a certain extent, which should be attributed to the low-temperature and high-salt marine environment where *Photobacteria* live (Moi et al., 2017). Notably, compared with PB_3262, PB_3285 shows a stronger appetite for salt and Ca^2+^, which may be due to the difference in their location in the cell. Based on their gene sequences, PB_3285 is predicted to contain a signal peptide and might be secreted extracellularly, but PB_3262 is not.

PB_3262 shares quite high identity (85.51%) with the strict exolytic 4-*O*-sulfatase PB4SF (Wang et al., 2019b), but it shows efficient endolytic activity toward CS polysaccharides, which endows it with more extensive application value in structural and functional studies of CS/DS. In recent years, two endolytic 4-*O*-sulfatases, BT_3349 and VB4SF, have been identified from *Bacteroides thetaiotaomicron* (Ulmer et al., 2014) and *Vibrio* sp. FC509 (Wang et al., 2015a), respectively. Compared with BT_3349 from gut bacteria, the two marine bacteria-derived PB_3262 and VB4SF have higher homology. However, in terms of substrate selectivity, both BT_3349 and VB4SF can hydrolyze the 4-*O*-sulfate groups of the A, iA and E units inside CS/DS polysaccharides to varying degrees (Ulmer et al., 2014; Wang et al., 2015a) but PB_3262 shows a very high specificity to the A unit in CS polysaccharides. In addition, unlike VB4SF, which can remove only 65% of 4-*O*-sulfate groups in CS-A under harsh conditions, PB_3262 can efficiently hydrolyze A units in CS-A to O units completely, although its activity against CS-A polysaccharide is much lower than that against disaccharide ΔA. In contrast, A units in CS-E are partially hydrolyzed to O units, indicating that the existence of E units may affect the hydrolysis of neighboring A units by PB_3262. Similarly, the ineffective activity toward DS indicates that the epimerization of GlcUA to IdoUA is also one of the inhibitory factors of this enzyme. Undoubtedly, these unique properties endow PB_3262 with important application value in structural and functional studies of CS/DS.

To date, several GalNAc-6-*O*-sulfatases have been identified from bacteria and animals, but all of them belong to exolytic sulfatases, and most, such as GalNAc-6-*O*-sulfatase from *Bacteroides thetaiotaomicron* (Ulmer et al., 2014) and mammalian lysosomal 6-*O*-sulfatase (Tomatsu et al., 1991), can only act on monosaccharide GalNAc(6S). Chondro-6-sulfatase from *Proteus vulgaris* is the only one that can act on oligosaccharides to strictly release 6-*O*-sulfates from GalNAc residues located at the reducing ends (Yamagata et al., 1968; Seno et al., 1974; Sugahara and Kojima, 1996) but has not been recombinantly expressed successfully. In this study, by using various structure-defined disaccharides and tetrasaccharides with different sulfation patterns, including ΔC, ΔD, ΔD-C and ΔC-D, we demonstrated that PB_3285 has strict exolytic activity to hydrolyze 6-*O*-sulfate groups from GalNAc residues at the reducing ends, which is similar to that of chondro-6-sulfatase from *Proteus vulgaris*. Thus, as the first recombinantly expressed GalNAc-6-*O*-sulfatase that can act on oligosaccharides, PB_3285 should be a more useful tool for the structural characterization of CS oligosaccharides.

Previous studies on the crystal structure and catalytic mechanism have shown that CS/DS sulfatases are highly conserved in 3D structure and key amino acid residues from bacteria to humans (Bond et al., 1997; Lukatela et al., 1998; Boltes et al., 2001; Hernandez-Guzman et al., 2003; Wang et al., 2019b; Ndeh et al., 2020). Thus, the 3D structures of PB_3262 and PB_3285 were constructed by homologous modeling using their homologous enzymes VB4SF from *Vibrio* sp. FC509 and lysosome GalN6S from humans as templates, and the key roles of the predicted key residues around active sites were further verified by inactive mutation. Notably, compared with that of PB_3262 the modeling structure of PB_3285 is debatable due to its low homology with human lysosome GalN6S (Rivera-Colon et al., 2012). In fact, the authentic structure of GalNAc-6-*O*-sulfatase from bacteria remains to be clarified in future studies.

## Conclusion

In conclusion, the identification and characterization of two marine CS sulfatases, PB_3262 and PB_3285, not only enrich the CS/DS sulfatase database but also provide potentially useful tools for structural and functional studies CS/DS and editing the sulfation patterns of CS/DS oligo-/polysaccharides.

## Data Availability Statement

The datasets presented in this study can be found in online repositories. The names of the repository/repositories and accession number(s) can be found below: https://www.ncbi.nlm.nih.gov/genbank/, MZ358908; https://www.ncbi.nlm.nih.gov/genbank/, MZ358909.

## Author Contributions

LW analyzed the data, wrote the manuscript under the guidance of FL. QZ isolated the strains and sequenced the draft genome. DL prepared CS tetrasaccharides. LW, MD, and XX performed the research. WW visualized the data. FL and XY designed the research and wrote the manuscript. All authors have read and approved the final manuscript.

## Conflict of Interest

The authors declare that the research was conducted in the absence of any commercial or financial relationships that could be construed as a potential conflict of interest.

## Publisher’s Note

All claims expressed in this article are solely those of the authors and do not necessarily represent those of their affiliated organizations, or those of the publisher, the editors and the reviewers. Any product that may be evaluated in this article, or claim that may be made by its manufacturer, is not guaranteed or endorsed by the publisher.

## Footnotes

1
https://blast.ncbi.nlm.nih.gov/Blast.cgi

2
https://www.expasy.org/

3
https://swissmodel.expasy.org/
