# Supplementary material for: Identification and Action Patterns of Two Chondroitin Sulfate Sulfatases From a Marine Bacterium Photobacterium sp. QA16
Source: Front Microbiol. 2022 Jan 24;12:775124. doi: 10.3389/fmicb.2021.775124 (PMC8819143; doi:10.3389/fmicb.2021.775124)
Supplement: Supplementary file 1 [file Data_Sheet_1.pdf]

Supplementary Material

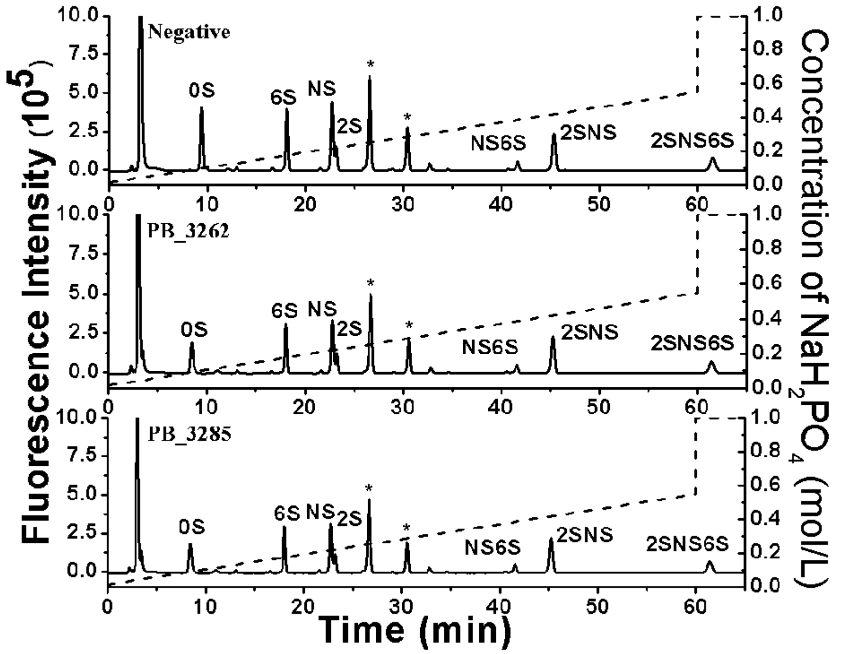

Figure S1. Product analysis of HS disaccharides treated with PB_3262 and PB_3285

To investigate the effects of PB_3262 and PB_3285 on unsaturated HS disaccharides, HS polysaccharide (3 µg) was exhaustively digested with heparinase II and an aliquot of the digest was treated without enzyme (top), with PB_3262 (middle) or with PB_3285 (bottom). The final products were further labeled with 2-AB and analyzed using YMC-Pack Polyamine II column eluted with a linear gradient from 0.016 to 0.55 M NaH_2PO_4 over a 60-min period. The elution positions of the standard disaccharides are indicated: 0S (ΔHexUA1–4GlcNAc), 6S (ΔHexUA1–4GlcNAc(6S)), NS (ΔHexUA1–4GlcNS), 2S (ΔHexUA(2S)1–4GlcNAc), NS6S (ΔHexUA1–4GlcNS(6S)), 2SNS (ΔHexUA(2S)1–4GlcNS), 2SNS6S (ΔHexUA(2S)1–4GlcNS(6S)). *, unidentified low-sulfated HS oligosaccharides.

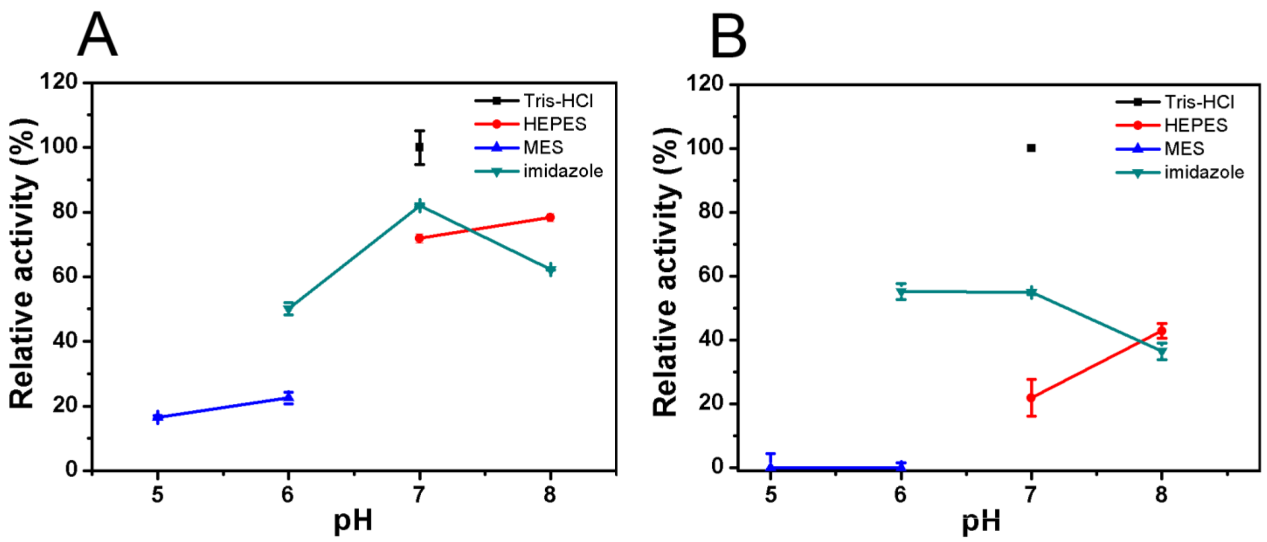

Figure S2. The effects of pH on PB_3262 and PB_3285 in other buffers

The activities of PB_3262 (A) and PB_3285 (B) in 50 mM HEPES, MES and imidazole buffers with different pH values from 5 to 8 were determined and presented as relative values calculated by comparing with the highest obtained in the 50 mM Tris-HCl buffer (pH 7.0).

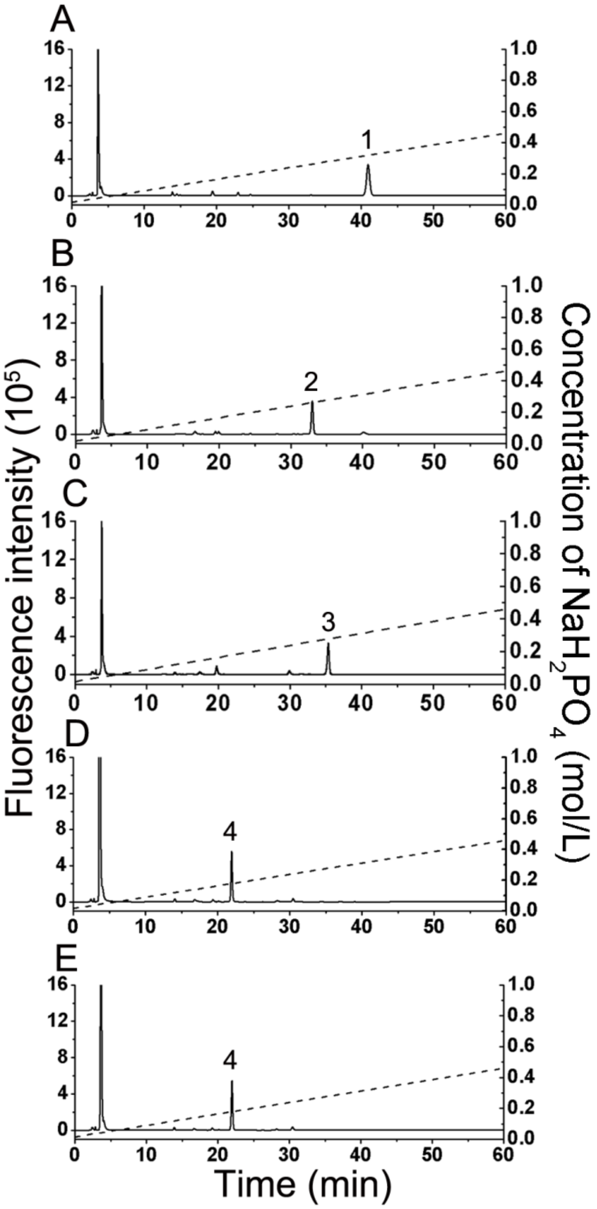

Figure S3. Activity analysis of PB_ 3285 against a saturated trisaccharide containing a non-reducing end GalNAc(6S) residue

A saturated trisaccharide GalNAc(6S)β1–4GlcUAβ1–3GalNAc with a non-reducing 6-*O*-sulfate was prepared by digesting ΔC-A with glycosyl hydrolase and PB_3262, and further treated with PB_3285 followed by 2-AB labeling and HPLC analysis as described under "*Materials and Methods*". A, tetrasaccharide ΔC-A; B, tetrasaccharide ΔC-A treated with PB_3262; C, tetrasaccharide ΔC-A treated with glycosyl hydrolase; D, tetrasaccharide ΔC-A treated with both PB_3262 and glycosyl hydrolase to remove ΔHexUA and 4-*O*-sulfate for preparing the saturated trisaccharide GalNAc(6S)β1–4GlcUAβ1–3GalNAc; and E, the prepared saturated trisaccharide GalNAc(6S)β1–4GlcUAβ1–3GalNAc treated with PB_3285. The elution positions of each peak are indicated:1, ΔHexUA1–3GalNAc(6S)β1–4GlcUAβ1–3GalNAc(4S); 2, ΔHexUA1–3GalNAc(6S)β1–4GlcUAβ1–3GalNAc; 3, GalNAc(6S)β1–4GlcUAβ1–3GalNAc(4S); 4, GalNAc(6S)β1–4GlcUAβ1–3GalNAc.

Table S1. Purification of recombinant PB_3262 and PB_3285

PB_3262				On ΔA		On CS-A	
	Total protein (g)	protein conc. (mg/ml)	Yield (%)	Total Activity (U)	Specific Activity (U/mg)	Total Activity (U)	Specific Activity (mU/mg)
Crude protein	0.668	4.7	100%	-	-	-	-
Elution from Ni^{2+} column	0.118	158.1	17.6%	19558	165.75	17.312	146.71

PB_3285				On ΔC		On ΔD		On ΔE	
	Total protein (g)	protein conc. (mg/ml)	Yield (%)	Total Activity (mU)	Specific Activity (mU/mg)	Total Activity (mU)	Specific Activity (mU/mg)	Total Activity (mU)	Specific Activity (mU/mg)
Crude protein	1.007	7.1	100%	-		-	-	-	-
Elution from Ni^{2+} column	0.165	220.0	16.4%	600.60	3.64	24.75	0.15	97.35	0.59

Table S2. Disaccharide compositions of CS/DS polysaccharides used as substrates

CS (mol%)	0S	4S	6S	2S6S	4S6S
CS-A	5	76	19	N.D.	N.D.
CS-D	2	31	49	18	N.D.
CS-E	5	42	17	N.D.	36
**DS (mol%)**	**0S**	**2S**	**4S**	**2S4S**	**4S6S**
DS	N.D.	5	88	7	N.D.

N.D. means not detected.
